# Metabolic Changes in Tumor Microenvironment: How Could They Affect γδ T Cells Functions?

**DOI:** 10.3390/cells10112896

**Published:** 2021-10-26

**Authors:** Anna Maria Corsale, Marta Di Simone, Elena Lo Presti, Carmela Picone, Francesco Dieli, Serena Meraviglia

**Affiliations:** 1 Department of Biomedicine, Neurosciences and Advanced Diagnosis, University of Palermo, 90133 Palermo, Italy; annamaria.corsale@unipa.it (A.M.C.); marta.disimone@unipa.it (M.D.S.); carmela.picone@unipa.it (C.P.); francesco.dieli@unipa.it (F.D.); 2 Central Laboratory of Advanced Diagnosis and Biomedical Research (CLADIBIOR), University of Palermo, 90127 Palermo, Italy; 3 National Research Council (CNR), Institute for Biomedical Research and Innovation (IRIB), 90146 Palermo, Italy; elena.lopresti@irib.cnr.it

**Keywords:** tumoral metabolism, γδ T cells, tumor microenvironment

## Abstract

The metabolic changes that occur in tumor microenvironment (TME) can influence not only the biological activity of tumor cells, which become more aggressive and auto sustained, but also the immune response against tumor cells, either producing ineffective responses or polarizing the response toward protumor activity. γδ T cells are a subset of T cells characterized by a plasticity that confers them the ability to differentiate towards different cell subsets according to the microenvironment conditions. On this basis, we here review the more recent studies focused on altered tumor metabolism and γδ T cells, considering their already known antitumor role and the possibility of manipulating their effector functions by in vitro and in vivo approaches. γδ T cells, thanks to their unique features, are themselves a valid alternative to overcome the limits associated with the use of conventional T cells, such as major histocompatibility complex (MHC) restriction, costimulatory signal and specific tumor-associated antigen recognition. Lipids, amino acids, hypoxia, prostaglandins and other metabolic changes inside the tumor microenvironment could reduce the efficacy of this important immune population and polarize γδ T cells toward IL17 producing cells that play a pro tumoral role. A deeper knowledge of this phenomenon could be helpful to formulate new immunotherapeutic approaches that target tumor metabolisms.

## 1. Introduction

It is well known that metabolic alterations related to cancer development are part of the “hallmarks of cancer” [1]. Cancer cells can modify the surrounding stroma by acidifying the environment [2,3], competing with other healthy cells for nutrients [4,5] and causing oxygen deprivation and reactive oxygen intermediates production due to accelerated metabolism required for tumor proliferation [6].

To date, an increased number of studies focus on the role of tumor cell metabolism [7]. The predominant metabolism program of tumor cells is the so-called “Warburg effect”, namely, their preferential use of aerobic glycolysis rather than oxidative phosphorylation (OXPHOS) to generate ATP, even in the presence of oxygen [8,9]. As a result, large glucose consumption implies the accumulation of lactic acid and consequently its release in the extracellular environment. Here, lactate induces the acidification of TME, improving tumor cells aggressiveness and negatively influencing the antitumor immune response [10]. In addition, tumor growth requires additional metabolic processes such as glutaminolysis or increasing lipogenesis. Glutamine metabolism provides amino acids, nucleic acids and glutathione, all of them necessary for tumor cell proliferation, thus depriving them of immune cells in the TME and limiting their ability to proliferate.

**Figure 1:**
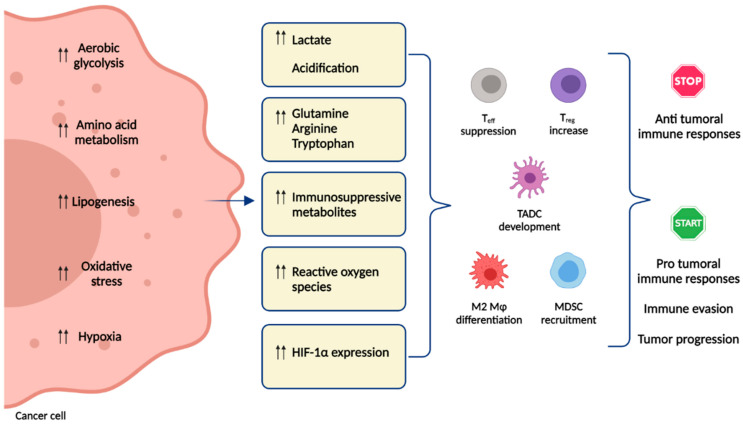
Implications of tumor metabolic reprogramming on immune responses. The immune response is affected by metabolic reprogramming in the TME and is driven by the increased demand for energy sources (glucose) and nutrients (glutamine, arginine, tryptophan) by cancer cells. Tumor-derived metabolites, such as lactate, prostaglandins, kynurenines, fatty acids, adenosine or reactive oxygen species, inhibit T_eff_ cell proliferation and cytotoxicity, turning off anti tumoral immune responses. Several immunosuppressive cells are activated or recruited to the TME (e.g., T_reg_, M2 Mφ, MDSC, TADC), favoring tumor progression. Double arrows indicate the upregulation of metabolic pathways in tumor cells. Abbreviations: T_eff_, effector T cells; T_reg_, regulatory T cells; M2 Mφ, activated M2 macrophages; MDSC, myeloid-derived suppressor cells; TADC, tumor-associated dendritic cells.

Moreover, tumor cells synthesize de novo fatty acids and cholesterol for plasma membrane biogenesis. High levels of these lipid metabolites in the TME steer immune cells toward immunosuppressive and anti-inflammatory functions [11]. In particular, cholesterol produced by tumor cells promotes the expression of suppressive immune checkpoints on T cells, causing their exhaustion and dysfunctional state [12]. As already mentioned, these metabolic changes have an influence on the immune response against tumor cells producing ineffective responses [13] or polarizing the response toward protumor activity [14,15] (Figure 1). Generally, immune cells under resting or activated states dictate the engagement of distinct metabolic pathways to proliferate and maintain effector functions. Naive T cells rely on the full oxidation of glucose through OXPHOS and fatty acid oxidation (FAO) to support ATP requests. It is known that upon TCR triggering by peptide antigen MHC, metabolic reprogramming occurs, mediated through the PI3K/Akt/mTOR pathway which increases glycolytic activity in T cells, similar to the “Warburg effect” seen in cancer cells [16,17]. Increased aerobic glycolysis, glutamine catabolism and fatty acid synthesis are observed in several subsets of effector T cells, including Th1, Th2, Th17 and cytotoxic CD8^+^ T cells and sustain their effector cytokine production and cytotoxic function. In contrast, memory and regulatory T cells are dependent upon FAO and OXPHOS pathways as naive T cells [16,17].

**Figure 2:**
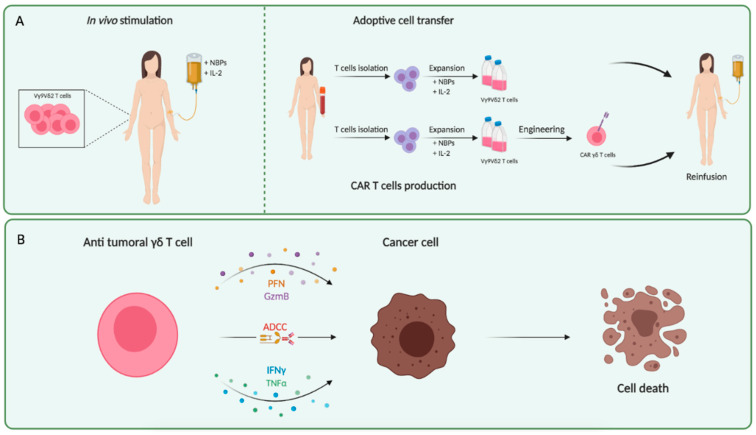
γδ T cells: clinical use and antitumor activity. (**A**) Clinical trials based on γδ T cells rely on the Vγ9Vδ2 subset, taking advantage of two distinct approaches, in vivo and ex vivo. In vivo stimulation involves N-BPs administration, in combination with IL-2, necessary for Vγ9Vδ2 expansion. Adoptive cell transfer (ex vivo approach) requires isolating T cells from PBMCs and Vγ9Vδ2 activation by culture with N-BPs plus IL-2. Then, the expanded Vγ9Vδ2 cells are reinfused into patients. Furthermore, to perform CAR-T cell production, Vγ9Vδ2 are expanded as previously and transduced with the specific CAR retrovirus. (**B**) The cytotoxic mechanisms exerted by γδ T cells, triggered upon TCR-dependent antigen recognition, mainly include perforin–granzyme pathway, ADCC, IFN-γ and TNF-α production. Abbreviations: N-BPs, nitrogen-containing bisphosphonates; IL-2, interleukine-2; PBMCs, peripheral blood mononuclear cells; CAR, chimeric antigen receptor; TCR, T cell receptor; ADCC, antibody-dependent cell-mediated cytotoxicity.

Although we know more about αβ T cell metabolism, there is weak knowledge on γδ T cells, which are also involved in antitumor immune response. Since the altered metabolism of tumor cells influences the environment in which also γδ T cells are recruited, a deeper knowledge of their energy metabolism could be helpful to improve γδ T cell-based immunotherapies [18,19,20]. A growing amount of evidence supports the idea that γδ T cells are involved in immune responses in the tumor microenvironment [21], although their role is still unclear, given that in different tumors they seem to correlate with positive [22] or negative [23,24] prognostic factors, or even do not correlate at all with patients’ outcome. Despite this, γδ T cells are considered excellent candidates for cellular immunotherapy against cancer cells as in the most avantgarde immunological approaches (CAR-T) [25] (Figure 2). Table 1 summarizes the main characteristics that make γδ T cells good defenders against tumors compared to others T cells [26,27].

γδ T cells are found either in tissues as part of the immune-resident population, or in the peripheral blood, as circulating cells. Tissue-resident γδ T cells preferentially express the Vδ1 chain frequently associated with different Vγ elements. On the other hand, circulating γδ T cells preferentially express the Vδ2 chain paired with the Vγ9 chain and this subset, which is referred to as Vγ9Vδ2, accounts for 50% to 90% of all γδ T cells [28,29].

Combined expression of differentiation markers such as CD45RA and CCR7 or CD27 allows distinguishing four different cellular types linked to their different functional capacity [30]. Naive cells express both markers and have a high proliferative capacity, shared with the central memory cells, which express only CCR7 or CD27. Instead, the cells that exert the effector and cytotoxic activities are effector memory (double negative) and terminally differentiated (CD45RA^+^), respectively, currently the best candidates for use in immunotherapies [31].

Ex vivo and in vitro studies have described the chameleon-like aspect of γδ T cells, which can change their functionality adapting to the microenvironment [32,33,34,35,36]. This aspect has been seen in our study on colorectal cancer [37] and squamous cell carcinoma [38], in which molecules produced by tumor cells or tumor-associated fibroblasts modify their proliferative and functional capacities. The microenvironment heterogeneity can be the bearer of changes that can damage the antitumor functions of γδ T cells, as pointed out in a study on breast cancer or hepatocellular carcinoma in which tumor-infiltrating γδ T cells correlated with the presence of IL17 and therefore with advanced stages of cancer [23,24].

## 2. Metabolic Features of γδ T Cells

As previously described, the activation and differentiation of immune cells are correlated to their metabolic profile, which represents a read-out key of functional states [39]. Recently, using SCENITH (Single Cell Metabolism by Profiling Translation Inhibition) protocol [40], Lopes and colleagues explored the distinct metabolic features of mouse γδ T cell subsets: antitumoral IFN-γ-producing γδ T cells (γδ^IFN^ cells) and protumoral IL17-producing γδ T cells (γδ^17^ cells) [41].

This flow-cytometry-based method allows analyzing energetic cell metabolism, monitoring changes in protein synthesis through the incorporation of puromycin. Next, with specific metabolic inhibitors, it is possible to estimate glucose and mitochondrial dependence, glycolytic capacity, fatty acid and amino acid oxidation capacity [40].

The authors demonstrated that, during thymic development, γδ T cells acquired specific metabolic profiles, preserved in peripheral lymphoid organs and TME of mouse breast and colon cancer models. In the periphery, γδ^17^ cells showed higher mitochondrial mass and membrane potential compared to γδ^IFN^ cells. According to this evidence, whereas antitumoral γδ^IFN^ cells showed high glycolytic activity, similarly to NK [42] and CD8^+^ T cells [43], γδ^17^ cells required OXPHOS-based mitochondrial metabolism, like Th17 cells [44].

Furthermore, evaluating mRNA levels of key mitochondrial and glycolysis-associated genes in purified peripheral γδ^17^ and γδ^IFN^ cells, they assessed that this metabolic duality had a transcriptional basis. On the one hand, γδ^17^ cells were characterized by the presence of Nrf, a regulator of mitochondrial DNA transcription, on the other hand, γδ^IFN^ cells expressed Myc, a regulator of glycolysis. Then, evaluating metabolic programs in the thymus, the authors showed that γδ thymocyte subpopulations followed the same trend seen in the periphery [41].

The following paragraphs will deal with the influence of metabolic intermediates on γδ T cells function and how the modulation of tumor metabolism can be exploited to boost the antitumor activity of γδ T cells for immunotherapy approaches.

## 3. Phosphoantigens and Nitrogen-Containing Bisphosphonates

An efficient anti-tumor response by γδ T cells relies on their recognition of tumor antigens. Vδ1 and Vδ2 subsets stand out for their ability to recognize distinct antigens. Although it is still not clear how Vδ1 activation occurs, it is known that they recognize MHC-related molecules, such as MICA, MICB and ULPB, commonly expressed on tumor cells [45,46]. Conversely, Vδ2 cells are activated by phosphoantigens (pAgs), a class of small lipidic molecules derived from the mevalonate pathway [47,48], without MHC restriction [49]

Examples of pAgs are isopentenyl pyrophosphate (IPP), which originates from the endogenous mevalonate (MVA) pathway, and (E)-4-Hydroxy-3-methyl-but-2-enyl pyrophosphate, an intermediate of the non-mevalonate pathway [50,51].

Hyperactive mevalonate metabolism, typical of tumor cells, alerts the immune system. The mevalonate pathway is upregulated in tumor cells, often due to a gain of function mutation of the oncosuppressor p53, which physiologically regulates this pathway [52]. Tumor cells may also undergo dysregulation of the MVA pathway due to losing control of HMG-CoA reductase or its over-expression [53]. All this determines an accumulation of mevalonate metabolites, including IPP, which activates the Vγ9Vδ2 TCR. As recently demonstrated, pAgs activation of Vγ9Vδ2 T cells critically depends on butyrophilin 3A1 and 2A1 [54,55,56,57,58]. IPP-activated Vγ9Vδ2 T cells can proliferate, produce effector cytokines such as IFN-γ and TNF-α and display natural killer cell-like cytotoxicity and thus represent a novel target of tumor immunotherapy [59].

Blocking the MVA pathway downstream, and consequently causing the accumulation of metabolites such as IPP, can be obtained using nitrogen-containing bisphosphonates (N-BPs), a type of drug currently used to treat osteoporosis and bone metastasis [60]. N-BPs inhibit farnesyl diphosphate synthase (FPPS), the enzyme that catalyzes the condensation between dimethyl-allyl-pyrophosphate and isopentenyl pyrophosphate to give rise to geranyl pyrophosphate, and the subsequent reaction with a further molecule of isopentenyl pyrophosphate to produce farnesyl pyrophosphate [61].

These data have been confirmed by the study of Li and colleagues using mevastatin (an IPP-synthesis-inhibiting drug) which reduced Vγ9Vδ2 T cells activation, while N-BP inhibition of FPPS promotes their stimulation [62].

Several years later, other studies demonstrated that myeloma, chronic lymphocytic leukemia and cancer stem cells become more susceptible to γδ T cells killing, when pretreated with N-BPs [63,64,65,66,67].

## 4. Lipid Metabolism

The primary metabolic resource of lipid metabolism is cholesterol, a fundamental component of membranes, because it regulates membrane fluidity and different receptor-mediated signal transduction pathways [68]. Many studies have shown a link between cholesterol homeostasis, cancer and immune response [69,70].

Cholesterol, LDL and HDL also influence the immunological activity of γδ T cells in the tumor context. A first study [71] identified two lipidic-related ligands of the Vγ9Vδ2 TCR on tumor targets: apolipoprotein A1 (Apo-A1) and ATP synthase/F1-ATPase (high-affinity apo A-I receptor) Apo-A1, abundant in HDL, are required for optimal activation of Vγ9Vδ2 T cells by tumors expressing F1-ATPase. Indeed, γδ T cell killing activity was impaired in the presence of Apo-A1-specific monoclonal antibody. Furthermore, the expression of the ATP synthase/F1-ATPase complex by tumor cells appears to influence the efficacy of killing of γδ T cells. Thus, the authors hypothesized that the Vγ9Vδ2 TCR, Apo-AI and F1 make a tri-molecular complex required for tumor cell recognition [71]. A second study [72] investigated whether γδ T cell immune response could be influenced by LDL uptake. The authors of that study found that Vδ2 T cells expressed LDL receptor upon activation and the binding of LDL caused alterations of Vδ2 T cells activation and functions. Notably, treatment of Vγ9Vδ2 T cells with LDL-cholesterol inhibited the expression of IFN-γ, NKG2D and DNAM-1.

Moreover, using an in vivo xenograft mouse model study, authors found that LDL-cholesterol treatment did not affect the level of γδ T cells in blood or tumor tissue, but γδ T cells appear less effective against tumor cells and failed to control tumor growth [72]. Thus, LDL-cholesterol uptake appears to act as an inhibitor of the antitumor functions of Vγ9Vδ2 T cells. Furthermore, upon other stress conditions, as during influenza infection, released host-derived lipids presented by lung infiltrating CD1d^+^B-1a cells, could activate γδ T cells for IL-17A induction via γδTCR-IRF4 pathway. Authors identified by single-cell RNA sequencing a subset of γδ^17^ cells with TCRγδ^hi^CD3^hi^AQP3^hi^CXCR6^hi^ phenotype, in both infected mice and patients with pneumonia [73]. Very recently, Lopes et al. [41] identified different metabolic programs of γδ T cell subsets, having a strong impact on their pro- and antitumoral activities in TME. They found that γδ^17^ cells are enriched by the uptake of lipids, such as palmidrate and cholesterol. γδ^17^ cells showed the ability to enhance tumor growth in tumor-bearing obese mice fed with the high-fat diet. The treatment with cholesterol increased their proliferation in vitro and promoted breast tumor growth upon adoptive cell transfer.

Conversely, in vitro supplementation of 10-fold higher glucose concentration augmented proliferation and cytotoxic activity of γδ^IFN^ cells against cancer cells, in addition to increased IFN-γ production and T-bet expression. Not coincidentally, after in vivo adoptive cell transfer of glucose-boosted γδ^IFN^ cells, a significant reduction of breast tumor development was observed. The same effect on proliferation status was not observed for γδ^17^ cells [41].

In light of these findings, there is an urgent need to study the involvement of lipid metabolism in the cytotoxic capacity of γδ T cells and, therefore, in their antitumor activity, to improve the efficacy of immunotherapies for those solid tumors against which the most modern clinical practices prove to be ineffective.

## 5. Amino Acid Metabolism

Within the TME, a competition is established between tumor cells and immune cells for amino acid requirements. Therefore, increased consumption of amino acids by tumor cells is necessary to sustain their constant growth and invasiveness [74], but at the same time, T cell activation requires amino acid uptake. Therefore, it is mediated by high expression of amino acid transporters, such as Large Neutral Amino Acids Transporter Small Subunit 1 (SLC7A5 also known as LAT1), Sodium-Dependent Neutral Amino Acid Transporter Type 2 (SLC1A5 also known as ASCT2) and Sodium- and Chloride-Dependent GABA Transporter 1 (SLC6A1 also known as GAT1) [75].

This duel was translated into compromised antitumor immune responses, inducing T cell anergy and affecting proliferation and effector activities [76].

The metabolism of L-arginine is one crucial regulator of immune responses and responsible for tumor progression. Multiple enzyme isoforms metabolize arginine: nitric oxide syntases (NOS1–3), arginases, glycine amidinotransferase and l-arginine decarboxylase [77]. NOS-mediated arginine metabolism gives rise to NO, which has strong immunomodulatory properties. NOS1 and NOS3 are constitutively expressed, instead, NOS2 is the inducible form [78] in response to pro-inflammatory cytokines such as IFN-γ, TNF-α, and IL-1β and bacterial lipopolysaccharide.

In a study by Douguet et al., NOS2 drives the polarization of γδ T cells toward a protumoral phenotype within mouse and human primary melanoma, inducing the production of IL17 and favoring metastatic progression result the recruitment of myeloid-derived suppressor cells (MDSC) [79]. The authors used mice transgenic for the Ret activated oncogene, which developed a spontaneous metastatic melanoma. In Ret mice knockout for NOS2, the correlation between the abundance of γδ T cells and MDSC was absent, compared to Ret mice, with impairing infiltration of γδ^17^ cells. Moreover, the presence of NOS2 reduced the killing capacity of γδ T cells against melanoma cell lines [79].

In a subsequent study, the authors confirmed that both cell populations are related to tumor progression [80]. Ret mice with a more aggressive melanoma showed a high infiltration of NOS2 expressing γδ T cells and were enriched in pro-inflammatory cytokines IL-1β and IL-6. Neutralization of these pro-inflammatory cytokines caused a reduction of the NOS2 enzyme produced by γδ T cells and a concomitant reduction of the tumoral aggressiveness, thus confirming an association between the expression of the enzyme by γδ T cells and tumor progression. In addition, in Ret mice with vitiligo the level of γδ T cells and MDSC proportionally decreased, as confirmed by confocal microscope images [80]. Hence, these findings suggest a crucial connection between IL17, γδ T cells and MDSC orchestrated by NOS2.

Finally, there is evidence that the presence of NOS2 also influences the metabolism of γδ T cells. NOS2-deficient mice showed a low abundance of γδ T cells in lymph nodes and a reduced capacity to produce IL-2. In addition, NOS2 neutralization inhibited γδ T cell proliferation and glycolysis, which was reversed by adding exogenous IL-2 [81].

Among other important amino acids, tryptophan is required for the survival and activation of T cells [82]. Its catabolism generates several metabolites (e.g., kynurenine, kynurenic acid, 3-hydroxy-kynurenine, and 3-hydroxy-anthranilic acid) released in the extracellular medium and are potentially toxic for immune cells. In particular, the kynurenines are overabundant in tumor cells that overexpress the enzymes indoleamine-2,3-dioxygenase (IDO) and tryptophan 2,3-dioxygenase, responsible of first reaction in the kynurenine pathway [83]. Tryptophan starvation and kynurenines abundance have been shown to exert immunoregulatory properties, such as hindering activation and proliferation of immune cells [84]. In the study by Jonescheit et al. [85], treatment with recombinant kynurenine decreased the cytotoxic capacity of Vγ9Vδ2 T lymphocytes towards ductal pancreatic adenocarcinoma cells. IDO inhibitors, 1-methyl-levo-trypthophan and 1-methyl-dextro-tryptophan improved Vγ9Vδ2 T lymphocyte cytotoxicity against pancreatic ductal adenocarcinoma (Panc89 and PancTu-I respectively) cell lines, after stimulation with bromohydrin diphosphate or in the presence of tribody [(HER2)_2_×Vγ9] [85].

## 6. Hypoxia and Metabolism

Tumors are characterized by the development of two types of hypoxia: chronic and cycling. Chronic hypoxia results from a persistent lack of oxygenation due to the inability of blood vessels to keep up with the growth rate of cancer cells, thus increasing the distance (>70 μm) between cells and blood vessels. Instead, cycling hypoxia, owing to unorganized vasculature, switches from episodes of oxygen reduction to re-oxygenation events [86].

Hypoxia-inducible factor 1α (HIF-1α) is the pivotal transcription factor of response to hypoxia and a key regulator of many genes that drive both types of hypoxia-triggered cellular mechanisms, such as cancer development and invasion and immunosuppression [87]. To fix insufficient oxygen supply, HIF-1α regulates the signaling of angiogenesis pathways and promotes vascularization of tumors, increasing the expression of proangiogenic factors such as vascular endothelial growth factor A, platelet-derived growth factor subunit A, transforming growth factor-β and angiopoietin-like 4 [88,89]. Solid tumors include hypoxic regions adjacent to normoxic areas that show significantly low [pO_2_] concentrations (<10 mmHg), compared to normal vascularized tissues [90]. Thus, hypoxia represents a negative prognostic factor and correlates to aggressiveness, metastatic progression and therapeutic resistance of tumors, as described in several reviews [91,92,93]. Indeed, HIF-1α regulates the expression of specific genes involved in the invasion, as fibronectin 1, lysyl oxidase-like 2, urokinase plasminogen activator receptor, epithelial–mesenchymal transition-associated transcription factors ZEB, SNAIL and TWIST [87].

Not coincidentally, intratumoral hypoxia supports immune evasion and cancer progression, inducing metabolic changes within TME. It underlies acidosis and high concentrations of lactate, adenosine and ammonium in the extracellular medium, in addition to nutrient deprivation and increase of heat shock protein expression [94].

On the one hand, these adaptations allow tumor cells to survive and proliferate in hypoxic TME. On the other hand, they impact anti-tumor immune response, resulting in decreased proliferation and effector functions of cytotoxic CD8^+^ T cells, CD4^+^ helper T cells, NK cells, M1 macrophages and dendritic cells, and support the recruitment of immunosuppressive cells, such as MDSC, M2 macrophages and T_reg_ cells [95,96]. At present, the knowledge about the influence of hypoxia on γδ T cells functions in TME is still controversial [97]. Hereafter, we comment on those few studies that illustrate this controversy.

A first study [98] investigated how hypoxia (1% O_2_) impacts the effector functions of γδ T cells in advanced oral cancer patients (III stage and IV stage). Expression of HIF1-α was increased in infiltrating Vδ2^+^ T cells compared to CD4^+^ and CD8^+^ T cells. However, upon TCR stimulation, the expansion, proliferation and activation status of circulating γδ T cells, purified from healthy donors, were not hindered under hypoxic conditions. Moreover, after 24 h of stimulation and under either hypoxia or normoxia (21% O_2_), γδ T cells secreted similar levels of IFN-γ, TNF-α and IL-6. However, when exposed to hypoxia, γδ T cells, expanded from healthy donors and oral cancer patients, significantly reduced their cytotoxic activity against N-BP-treated oral cancer cell lines compared to normoxia.

Further, this ability was reduced when tumor target cells were exposed themselves to hypoxia. Thus, hypoxia compromised anti-tumor cytotoxicity of γδ T cells due to a reduction of calcium efflux and CD107a expression, which are responsible for regulating their effector functions. In addition, the authors assessed the impact of hypoxia on the differentiation of γδ T cells. Under hypoxic conditions, γδ T cells differentiated towards IL17A-producing γδ T cells, showing elevated RORγt expression. Consistent with these results, γδ^17^ cells were increased among tumor-infiltrating lymphocytes in advanced oral cancer patients [98].

Park et al. showed that γδ T cells in the glioma TME express high levels of HIF-1α and Bax but reduced cytotoxic activity [99]. As brain tumors ran out of a lot of oxygen compared to other cancers and took it away from TILs, the authors speculated that inhibiting hypoxia could restore the ability of γδ T cells to kill tumor cells. In particular, hypoxia-mediated activation of the cyclic AMP-PKA signaling pathway in γδ T cells reduced NKG2A receptor expression, thereby abolishing their cytotoxicity. As previously demonstrated, upon metformin treatment, the oxygen uptake by tumor cells was reduced and more oxygen was available for TILs. Accordingly, following metformin treatment, antitumor γδ T cells were restored, given by NKG2A and CD107a upregulation, granzyme B and IFN-γ production, and acquired the ability to resist apoptosis [100]. Thus, the treatment of ex vivo expanded human γδ T cells with metformin or HIF-1α inhibitor showed roughly 75% tumor-free survival in the glioma xenograft model [99]. In their study to evaluate the effects of hypoxia on cytotoxic functions of γδ T cells, Siegers et al. established in vitro co-cultures between γδ T cells and breast cancer cell lines under hypoxic condition. When γδ T cells were pre-incubated for 48 h in hypoxia, they exerted enhanced cytotoxicity against breast cancer cell lines, but breast tumor cell lines pre-cultured in hypoxia became resistant to γδ T cells killing due to MICA/B shedding by breast cancer cells [101].

Finally, in another recent paper, hypoxia attenuated antitumor effector functions of γδ T cells, enhanced by oral squamous cell carcinoma-derived exosomes. This effect was related to the hypoxia-mediated suppressive effect of MDSCs on γδ T cells through a miR-21/PTEN/PD-L1 regulation axis [102].

## 7. COX-2 and Prostaglandins

Tumors often overexpress the cyclooxygenase 2 (COX-2) enzyme that catalyzes the metabolism of arachidonic acid to prostanoids, such as prostaglandins and thromboxanes [103].

Prostaglandin E2 (PGE2) binds four G-protein-coupled EP receptors (EP1–EP4) to improve tumor cell growth and survival, promote angiogenesis and induce metastatic cascade [104]. Thus, it actively contributes to establishing an immunosuppressive milieu playing a dual role: on one hand, PGE2 inhibits macrophages, neutrophils and Th1, NK and cytotoxic T cells; on the other hand, it promotes the activities of suppressive immune cells, such as T_reg_, Th2, Th17 and MDSC [105].

In COX-2-expressing pancreatic ductal adenocarcinoma, increased levels of PGE2 induced resistance against γδ T cell cytotoxicity, which was restored by the co-treatment with COX-2 inhibitor DuP697 and tribody [(Her2)2×Vγ9] [106]. Furthermore, PGE2-induced inhibition is mediated through activation of protein kinase A type I and interlopes with the cytotoxic receptors of γδ T cells (CD16, NKG2D and TCR Vγ9Vδ2) [107]. Not by chance, the crucial role of PGE2 was confirmed in experiments assessing the immunosuppressive activity of mesenchymal stem cells against pAg-activated Vγ9Vδ2 T cells [108]. The release of inflammatory cytokines, such as IFN-γ and TNF-α, by γδ T cells induced higher expression of COX-2 enzyme in mesenchymal stem cells with a consequent PGE2 production. In turn, PGE2 bound to G-coupled EP2 and EP4 receptors expressed on γδ T cells and increased cyclic adenosine monophosphate levels via adenylate cyclase activation. Thus, mesenchymal stem cells suppressed proliferation, cytokine production and cytotoxic ability of γδ T cells with negative feedback [108].

## 8. Conclusions

**Figure 3:**
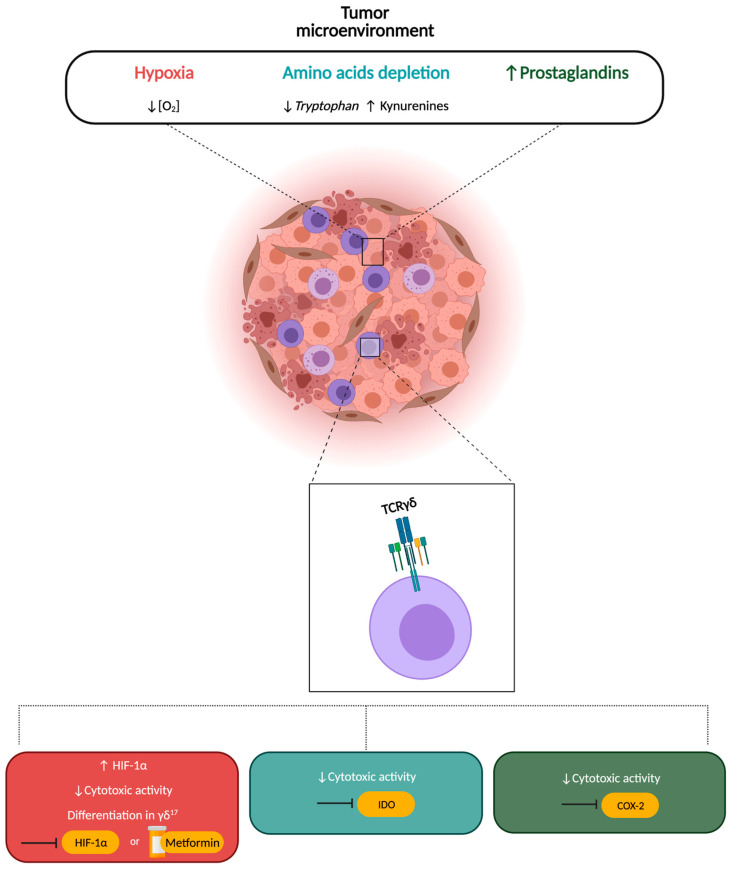
Strategies targeting metabolic alterations in the TME to improve γδ T cell effector functions. The competition for crucial nutrients, the accumulation of toxic molecules and a hypoxic environment hamper anti-tumor immune responses played by γδ T cells, impairing their cytotoxic activity or inducing their differentiation into γδ^17^ with protumor functions. Conversely, manipulating tumor metabolism with specific inhibitors of HIF-1α, IDO or COX-2 or with the use of metformin inhibits these metabolic pathways and restores immune cells response. Abbreviations: γδ^17^, IL17-producing γδ T cells; HIF-1α, hypoxia-inducible factor 1-alpha; IDO, indoleamine-pyrrole 2,3-dioxygenase; COX-2, cyclooxygenase isoenzyme-2. Down arrow: low, up arrow: high.

Since our and other authors’ studies show that the TME alters the functions of γδ T cells [37,38], there is a need for more in-depth studies to define the molecules and the metabolic pathways responsible. In addition, these studies should answer some fundamental questions: (1) Do γδ T cells with pro- and antitumor activities have a distinct metabolic pattern in different types of cancer? (2) Can the TME contain specific metabolic biomarkers which orchestrate selective programs in γδ T cells? (3) More generally, do particular metabolites in the TME modify innate and adaptive immune responses? Overall, these studies should provide comprehensive information, which should then be exploited to develop novel γδ T cell-based therapeutic strategies [109] (Figure 3).

## Figures and Tables

**Table 1:** Characteristics that make γδ T cells good defenders against tumors compared to others T cells.

Features	γδ T Cells	Other T Cells
High frequency	✗	✓
Recognize and lyse a broad range of tumor cells	✓	✓
Lack MHC restriction in antigen recognition	✓	✗
Require co-stimulatory signals (e.g., CD28)	✗	✓
Cytotoxic abilities (ADCC)	✓	✗
In vivo activation using FDA-approved drugs (Zoledronate, IL-2)	✓	✗

## Data Availability

Not applicable.
